# Overdose from Unintentional Fentanyl Use when Intending to Use a Non-opioid Substance: An Analysis of Medically Attended Opioid Overdose Events

**DOI:** 10.1007/s11524-024-00852-0

**Published:** 2024-04-03

**Authors:** Alexander R. Bazazi, Patrick Low, Bryson O. Gomez, Hannah Snyder, Jeffrey K. Hom, Christine S. Soran, Barry Zevin, Michael Mason, Joseph Graterol, Phillip O. Coffin

**Affiliations:** 1 Division of Substance Abuse and Addiction Medicine at San Francisco General Hospital, Department of Psychiatry and Behavioral Sciences, University of California San Francisco, 1001 Potrero Ave, San Francisco, CA 94110 USA; 2 Department of Family and Community Medicine, University of California San Francisco, 995 Potrero Ave, San Francisco, CA 94110 USA; 3 Population Behavioral Health, Behavioral Health Services Division, San Francisco Department of Public Health, 101 Grove St, San Francisco, CA 94102 USA; 4 Street Medicine and Shelter Health, San Francisco Department of Public Health, 555 Stevenson St, San Francisco, CA 94105 USA; 5 Community Paramedicine Division, San Francisco Fire Department, 698 2nd St, San Francisco, CA 94107 USA; 6 Division of General Internal Medicine at San Francisco General Hospital, Department of Medicine, University of California San Francisco, 1001 Potrero Ave, San Francisco, CA 94110 USA; 7 Division of HIV, Infectious Diseases and Global Medicine, Department of Medicine, University of California San Francisco, 505 Parnassus Avenue, San Francisco, CA 94143 USA; 8 Center On Substance Use and Health, San Francisco Department of Public Health, 101 Grove St, San Francisco, CA 94102 USA

**Keywords:** Fentanyl, Opioid, Stimulant, Overdose, Emergency medical services

## Abstract

Fentanyl-mixed and substituted heroin is well-documented, but less is known about unintentional fentanyl use among people using stimulants. To determine the prevalence of and racial and ethnic disparities in unintentional fentanyl use among people experiencing a medically attended opioid overdose, we reviewed 448 suspected non-fatal overdose cases attended by a community paramedic overdose response team in San Francisco from June to September 2022. We applied a case definition for opioid overdose to paramedic records and abstracted data on intended substance use prior to overdose. Among events meeting case criteria with data on intended substance use, intentional opioid use was reported by 57.3%, 98.0% of whom intended to use fentanyl. No intentional opioid use was reported by 42.7%, with most intending to use stimulants (72.6%), including methamphetamine and cocaine. No intentional opioid use was reported by 58.5% of Black, 52.4% of Latinx, and 28.8% of White individuals (*p* = 0.021), and by 57.6% of women and 39.5% of men (*p* = 0.061). These findings suggest that unintentional fentanyl use among people without opioid tolerance may cause a significant proportion of opioid overdoses in San Francisco. While intentional fentanyl use might be underreported, the magnitude of self-reported unintentional use merits further investigation to confirm this phenomenon, explore mechanisms of use and disparities by race and ethnicity, and deploy targeted overdose prevention interventions.

**Supplementary Information:**

The online version contains supplementary material available at 10.1007/s11524-024-00852-0.

## Introduction

In 2022, opioid overdoses caused over 70,000 deaths in the U.S. and 649 deaths in San Francisco, with 91% attributable to fentanyl and related analogues [1–3]. Nationally and locally, opioid overdose mortality has disproportionally affected Black individuals, who experience overdose deaths with alarming frequency [2, 4]. At the same time, deaths involving stimulants (i.e. cocaine and methamphetamine) also attributed to fentanyl have become increasingly common nationwide [5]. Although exposure among people who use opioids to fentanyl-mixed or substituted heroin is well-documented [6, 7], less is known about unintentional fentanyl use among people who primarily use non-opioid substances. Even rare fentanyl exposure in individuals without opioid tolerance could meaningfully contribute to overdose mortality. Multiple lines of evidence highlight the potential risk of unintentional fentanyl use among people who use stimulants, including reports of opioid overdose clusters among people intending to use stimulants [8, 9], as well as drug testing services and seizure data showing fentanyl in stimulant samples [10, 11]. Despite documented concern from people who use drugs [12], there is a paucity of research directly assessing unintentional opioid use in individuals intending to use stimulants or other non-opioid substances [13].

In San Francisco, the impact of unintentional fentanyl use on fentanyl-attributed deaths also remains unclear. Findings from a community-based study of women experiencing homelessness in San Francisco suggest that the presence of fentanyl in substances thought to be stimulants may be a rare occurrence [14]. Yet a study of fentanyl and stimulant overdose decedents in San Francisco revealed significantly less chart-documented pre-mortem opioid use than expected, suggesting that a significant proportion of fentanyl-stimulant poisoning decedents may not have regularly used opioids prior to their death [15]. This evidence and increasing reports from staff on post-overdose response teams of clients endorsing unintentional fentanyl use motivated the current investigation. We used data from a case review of first-responder records from individuals who experienced a non-fatal opioid overdose in San Francisco to estimate the prevalence and demographic correlates of unintentional opioid use overdose.

## Methods

### Data Source

We reviewed consecutive records of suspected overdoses from June to September 2022 from San Francisco’s Street Overdose Response Team (SORT). SORT is a specialized post-overdose response team consisting of community paramedics and peer counsellors attending to suspected overdose cases often alongside general emergency medical services (EMS). Case records included both structured and unstructured data. Structured data included demographics, behavioral activity rating scale (BARS) score [16], and clinical opiate withdrawal scale (COWS) score [17], which was only used in a sensitivity analysis due to reliability concerns given variability in the timing and dosing of naloxone and time of assessments. Unstructured data abstracted from paramedic-recorded free text notes included reports of overdose, patient responsiveness, naloxone administration, naloxone response, and patient-reported intended substance used prior to overdose. Data were imported into *REDCap *where unstructured data were manually abstracted by the research team [18].

### Overdose and Unintentional Opioid Use Determination

The case definition for opioid overdose was based on the initial clinical presentation and subsequent improvement in responsiveness or respiratory rate after naloxone administration (Table 1). Our case definition was adapted from the Rhode Island Criteria, a validated approach for opioid overdose determination from EMS data [19], though since our clinical impression data were unstructured, the definition required modification. As a sensitivity analysis, we piloted an alternative case definition that also utilized BARS and COWS scores to identify increased responsiveness after naloxone administration. Intended substance used and intentionality of opioid use were determined based on patient self-report, recorded in unstructured text responses. Two members of the research team independently reviewed records in sets of 20 until achieving > 95% concordance on opioid overdose determination, intended substance used, and intentionality of opioid use, after which reviews were independently conducted by one individual, with a random subset of 5% verified by the principal investigator.

**Table 1:** Opioid overdose criteria

Narrative includes at least 1 of the following: • report of suspected overdose to dispatcher • unresponsiveness to verbal or physical stimuli (per bystander or EMS)	**AND**	**Naloxone administration:** Administered by either EMS or bystander prior to EMS arrival	**AND**	**Symptom improvement:** Narrative includes at least 1 of the following: • Responsive to verbal or physical stimuli • Improvement in respiratory rate

### Analysis

We calculated the proportion of opioid overdoses attributed to intentional and unintentional opioid use and compared demographics. Among those reporting no intentional opioid use, we examined which substances people intended to use. All statistical analyses were conducted in *R, version 4.3*.2 [20].

### Ethical Approval

This study was reviewed and deemed exempt by the University of California, San Francisco Institutional Review Board (IRB #23–40491).

## Results

Out of 448 response records for suspected overdose, 281 met the case definition for opioid overdose and 171 also included data on intended substance use at the time of overdose. Of these 171 cases, 98 (57.3%) reported intentional opioid use and 73 (42.7%) reported no intentional opioid use at the time of overdose. Among those intending to use opioids, 96 (98.0%) reported intending to use fentanyl and only two (2.0%) reported intending to use heroin. Among those not intending to use opioids, 53 (72.6%) intended to use stimulants, including methamphetamine (43.8%), crack cocaine (15.1%), powder cocaine (11.0%), and unspecified stimulant (2.7%). Fifteen individuals (20.5%) reported cannabis use and 5 (6.8%) reported some other substance (3 alcohol, 1 GHB, 1 tobacco).

With regard to demographics (Table 2), intentionality of opioid use differed by race and ethnicity, with no intentional opioid use reported by 58.5% of Black, 52.4% of Latinx, and 28.8% of White individuals (*p* = 0.021). By gender, 57.6% of women and 39.5% of men reported no intentional opioid use (*p* = 0.061). Those reporting no intentional opioid use were older than those reporting intentional opioid use (median age 40.5 years vs. 36.0 years, *p* = 0.043).

**Table 2:** Unintentional and intentional opioid exposure by survivor demographics and intended substance use

	Overall (*n*, % of total sample)	Unintentional opioid (*n*, % of subgroup with outcome)	Intentional opioid (*n*, % of subgroup with outcome)	*P*-value
Total	N = 171	N = 73	N = 98	
**Race and ethnicity**				0.021^a^
Asian	5 (2.9%)	2 (40.0%)	3 (60.0%)	
Black or African American	41 (24.0%)	24 (58.5%)	17 (41.5%)	
Hispanic or Latinx	21 (12.3%)	11 (52.4%)	10 (47.6%)	
White	66 (38.6%)	19 (28.8%)	47 (71.2%)	
Other race/ethnicity	15 (8.8%)	8 (53.3%)	7 (46.7%)	
Missing	23 (13.5%)	9 (39.1%)	14 (60.9%)	
**Gender**				0.061^b^
Women	33 (19.3%)	19 (57.6%)	14 (42.4%)	
Men	129 (75.4%)	51 (39.5%)	78 (60.5%)	
Non-Binary	1 (0.6%)	1 (100%)	0 (0%)	
Missing	8 (4.7%)	2 (25.0%)	6 (75.0%)	
**Age**				0.043^c^
Mean, y (SD)	40.2 (13.0)	42.7 (14.2)	38.3 (11.8)	
Median, y [Min, Max]	38.0 [19.0, 77.0]	41.0 [21.0, 77.0]	36.0 [19.0, 73.0]	
	Overall (*n*, % of total sample)	Unintentional opioid (*n*, % of outcome)	Intentional opioid (*n*, % of outcome)	
**Intended substance**				
Fentanyl	96 (56.1%)	0 (0%)	96 (98.0%)	
Heroin	2 (1.2%)	0 (0%)	2 (2.0%)	
Any stimulant	53 (31.0%)	53 (72.6%)	0 (0%)	
Methamphetamine	32 (18.7%)	32 (43.8%)	0 (0%)	
Cocaine, crack	11 (6.4%)	11 (15.1%)	0 (0%)	
Cocaine, powder	8 (4.7%)	8 (11.0%)	0 (0%)	
Stimulant, unspecified	2 (1.2%)	2 (2.7%)	0 (0%)	
Cannabis	15 (8.8%)	15 (20.5%)	0 (0%)	
Other substance	5 (2.9%)	5 (6.8%)	0 (0%)	

^a^Fisher’s exact test after excluding individuals with missing race/ethnicity data; *p* = 0.042 when including missing data as race/ethnicity category. ^b^Fisher’s exact test after excluding individuals with missing gender data; *p* = 0.089 when including missing data as gender category. ^c^Wilcoxon rank-sum test

The alternative case definition that allowed for evidence of post-naloxone improvement in responsiveness from improvement in the BARS score or increase of the COWS score resulted in an additional 3 of 448 responses being classified as opioid overdoses, but none of these individuals had data on intended substance use for inclusion in the analysis, so results were unchanged. An additional analysis found similar demographics among those with missing compared to complete intended substance use data, although a greater proportion of those with missing substance use data had “other” or “missing” data on race and ethnicity (Supplementary Information).

## Discussion

In this first study to document the prevalence of self-reported unintentional opioid use among people who have just experienced an opioid overdose, we found that nearly half of overdoses were attributed to unintentional opioid use, with most of these reported to occur while intending to use stimulants, both cocaine and methamphetamine. In San Francisco, fentanyl is the opioid involved in over 91% of opioid overdose deaths and was the reported opioid used by 98% of individuals intending to use opioids in this cohort [3]. Unintentional opioid use is believed to be almost entirely attributable to fentanyl in San Francisco, given that fentanyl is the predominant street opioid and that the now dwindling heroin market remains dominated by black tar heroin that is easily distinguished from and rarely mixed with fentanyl due to physical characteristics, in contrast to street opioid markets in eastern U.S. states [3, 21, 22]. No individuals in this sample reported intending to use opioid or other pills or tablets.

This study does not identify potential mechanisms of unintentional fentanyl use, which include mistaking fentanyl for other substances (stimulants and fentanyl are both often sold as white powder or crystals), fentanyl contamination of non-opioid substances, storage of drugs together, and reuse of equipment previously used to consume fentanyl. Prior public health investigations have highlighted clusters of overdoses occurring among people mistakenly using fentanyl [8, 9], although the incidence of unintentional fentanyl use has not been studied in cohort studies or at the population level. A recent laboratory analysis of stimulants collected by community-based drug checking services across the country found that approximately 9% of samples thought by the donor to be methamphetamine or cocaine contained fentanyl, an issue that was more prevalent when powdered drugs were tested [10]. In San Francisco, drug checking services and urine surveillance in a community-based study suggest that the presence of fentanyl in substances thought to be stimulants is a rare occurrence [14], although even a rare occurrence could meaningfully contribute to mortality among people who use drugs daily. Regarding exposure through cross-contaminated equipment, to our knowledge no prior work has examined the potential for overdose from reusing smoking, sniffing, or injection equipment previously used to consume fentanyl.

Unintentional fentanyl use overdoses were significantly more prevalent among Black and Latinx individuals compared to White individuals, more prevalent among women compared to men, and more prevalent among older persons. Observed racial and ethnic disparities may relate to differences in drug use patterns, drug selling networks, or the impact of structural racism on other aspects of the overdose risk environment [23, 24], but observed disparities could also be related to underreporting of intentional fentanyl use due to stigma or perceived risk of disclosure among minoritized individuals. We find it unlikely, however, that underreporting alone could explain the magnitude of racial disparities observed. These findings suggest that efforts to prevent overdose from unintentional fentanyl use among people using stimulants, if directed toward minoritized communities, may help to lessen some of the profound disparities in overdose mortality.

Our study has several limitations. First, we relied upon self-report, which as discussed may overestimate the rate of unintentional fentanyl use due to underreporting of intentional use. Reports of intended use of cannabis may indicate such underreporting, as cannabis is legal for personal consumption in California and thus less likely to be underreported and is unlikely to be mistaken for or mixed with fentanyl; however, smoking cannabis in opioid cross-contaminated equipment could theoretically be a potential route of unintentional fentanyl exposure. Second, our case definition, adapted from an established definition for opioid overdose to accommodate available data, likely has imperfect sensitivity and specificity. Cases of suspected overdose not meeting the case definition either represented events that were not overdoses or overdose events with insufficient documentation to be classified as such. We modified the definition in a sensitivity analysis with no improvement in case capture. Missing data on intended substance use was common and likely attributable to altered mental status after overdose reversal or unwillingness to disclose, though even if all those missing intended substance use data represented intentional fentanyl use, our estimate of unintentional fentanyl use would still be remarkably high at 28% (78/281). It is possible that differential missingness of intended substance use data by race and ethnicity could have induced the observed association between race and ethnicity and unintentional opioid use. Finally, our results may not generalize beyond San Francisco due to the regional nature of street drug markets and use patterns, though given regional similarities, they may apply more to western U.S. states than eastern states.

In conclusion, we find preliminary evidence that unintentional fentanyl use may contribute substantially to opioid overdose in San Francisco. Attention to unintentional use, particularly among people who use stimulants, should be a priority for overdose prevention efforts and efforts to address racial disparities in overdose mortality. Further research should consider mixed methods approaches to characterize reported unintentional use, cohort studies to determine incidence, and exploration of pharmacotherapy for opioid blockade. Existing interventions including naloxone distribution, drug testing services, access to safer use equipment to reduce cross-contamination, and overdose prevention sites should be scaled up.

### Supplementary Information

Below is the link to the electronic supplementary material.

Supplementary file1 (DOCX 21 KB)
