# Supplementary material for: Overdose from Unintentional Fentanyl Use when Intending to Use a Non-opioid Substance: An Analysis of Medically Attended Opioid Overdose Events
Source: J Urban Health. 2024 Apr 3;101(2):245–51. doi: 10.1007/s11524-024-00852-0 (PMC11052958; doi:10.1007/s11524-024-00852-0)
Supplement: Supplementary file 1 — Supplementary file1 (DOCX 21 KB) [file 11524_2024_852_MOESM1_ESM.docx]

**Supplemental Materials**

**Table S1.** Demographic characteristics of opioid overdose cases reporting intended substance use (n=171) and those not reporting intended substance use (n=110).

|  | **Overall**  **(n, % of total sample)** | **Intended Substance Missing or Indeterminate (n, %)** | **Intended Substance Reported (n, %)** | **P-value** |
| --- | --- | --- | --- | --- |
| Total | N=281 | N=110 | N=171 |  |
| **Race and ethnicity** |  |  |  | 0.038^a^ |
| Asian | 5 (1.8%) | 0 (0%) | 5 (2.9%) |  |
| Black or African American | 57 (20.3%) | 16 (14.5%) | 41 (24.0%) |  |
| Hispanic or Latinx | 31 (11.0%) | 10 (9.1%) | 21 (12.3%) |  |
| White | 104 (37.0%) | 38 (34.5%) | 66 (38.6%) |  |
| Other race/ethnicity | 34 (12.1%) | 19 (17.3%) | 15 (8.8%) |  |
| Missing | 50 (17.8%) | 27 (24.5%) | 23 (13.5%) |  |
| **Gender** |  |  |  | 1.0^b^ |
| Women | 52 (18.5%) | 19 (17.3%) | 33 (19.3%) |  |
| Men | 201 (71.5%) | 72 (65.5%) | 129 (75.4%) |  |
| Non-Binary | 1 (0.4%) | 0 (0%) | 1 (0.6%) |  |
| Missing | 27 (9.6%) | 19 (17.3%) | 8 (4.7%) |  |
| **Age** |  |  |  | 0.211^c^ |
| Mean, y (SD) | 39.4 (12.4) | 38.1 (11.4) | 40.2 (13.0) |  |
| Median, y [Min, Max] | 37.0 [19.0, 77.0] | 37.0 [19.0, 71.0] | 38.0 [19.0, 77.0] |  |
|  |  |  |  |  |

^a^Fisher’s exact test after excluding individuals with missing race/ethnicity data; p=0.014 when including missing data as race/ethnicity category. ^b^Fisher’s exact test after excluding individuals with missing gender data; p=0.003 when including missing data as gender category. ^c^Wilcoxon rank-sum test.
